# *In vitro* Sequestration of Molecular and Mass Spectra Characterized Metallophilic Cadmium Tolerant Bacteria for Sustainable Agriculture

**DOI:** 10.3389/fmicb.2022.845853

**Published:** 2022-03-31

**Authors:** Baba Uqab, Ruqeya Nazir, Bashir Ahmad Ganai, Praveen Rahi

**Affiliations:** ^1^Department of Environmental Science, University of Kashmir, Srinagar, India; ^2^Center of Research for Development, University of Kashmir, Srinagar, India; ^3^National Center for Microbial Resource, National Center for Cell Science, Pune, India

**Keywords:** bacteria, heavy metals, MALDI-TOF MS, sequestration, 16S rRNA

## Abstract

Due to industrialization, the contamination of toxic metals in soils is currently one of the major concerns to scientists worldwide. The presence of high concentrations of heavy metals including cadmium in the environment is mainly attributed to human activities. Being a highly toxic metal, cadmium can enter plant cell transporters usually used for the uptake of essential cations, such as iron, calcium, and zinc. This study deals with the appraisement of response and tolerance shown by various bacteria in varied cadmium concentrations (100–1,000 ppm). The optical density (OD) of the isolates was measured to determine the minimum inhibitory concentration (MIC) of cadmium. Isolated bacteria have been identified using 16S rRNA gene sequence and Matrix-assisted laser desorption ionization time-of-flight mass spectrometry (MALDI-TOF MS). Among the 72 isolates, 07 (*Bacillus pumilus, Enterobacter kobei, Klebsiella pneumonia, Pseudomonas mandelii, Pseudomonas putida, Pseudomonas avellanae*, and *Staphylococcus equorum*), isolates had efficacy for cadmium tolerance and showed sequestration potential at varying MIC. Furthermore, *K. pneumonia* was observed to have the highest (900 ppm) tolerance for cadmium and the lowest (600 ppm) was shown by *E. kobei*. Besides, *K. pneumonia* showed the highest (75.2%) sequestration potential while the least (52.4%) potential was observed for *P. putida*. These cadmium tolerant species can be implemented in contaminated environments for detoxification and elimination of cadmium from these agricultural fields.

**Graphical Abstract:**
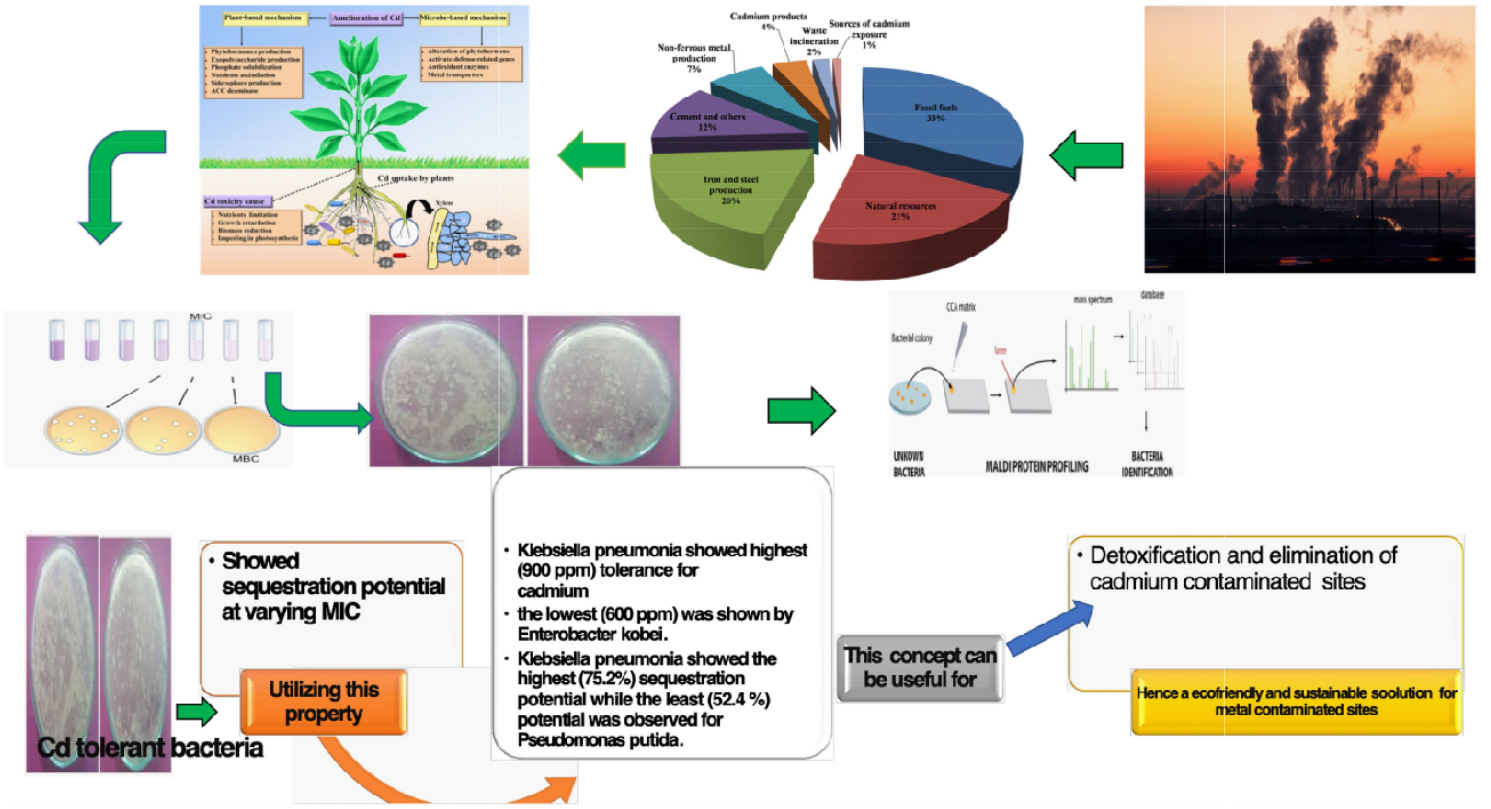

## Highlights

- 07 Bacterial isolates (*Pseudomonas putida, Bacillus pumilus, Klebsiella pneumonia, Pseudomonas mandelii, Pseudomonas avellanae, Enterobacter kobei*, and *Staphylococcus equorum*) were found to have efficacy for cadmium tolerance and showed sequestration potential at varying MIC.
- *Klebsiella pneumonia* was observed to have the highest (900 ppm) tolerance for cadmium and the lowest (600 ppm) was shown by *E. kobei*.
- *Klebsiella pneumonia* showed the highest (75.2%) sequestration potential while the least (52.4%) potential was observed for *P. putida*.
- All the isolated bacteria were identified using latest and robust techniques.

## Background

Heavy metal contamination aggravated by various human induced activities is responsible for serious environmental and ecological problems both in soil and water (Wong et al., 2006; Förstner and Wittmann, 2012; Qin et al., 2021). Increased heavy metal contamination of the natural environment, particularly in places under anthropogenic stress, also contributes to microbial population imbalances (Hrynkiewicz and Baum, 2014). In many terrestrial ecosystems, such as sediments (Yin et al., 2015; Li et al., 2017) and neutral mine drainage polluted soils, heavy metal contamination induced apparent changes in microbial community structure rather than microbial diversity (Pereira et al., 2014). The bacterial resistance to heavy metals results from key interactions in adapting with natural metals (Hall, 2002; Briffa et al., 2020). According to studies carried by Bouskill et al. (2010) the variation of the response of bacteria especially (Proteobacteria) to heavy metal may due to the phylum exhibits a complex lifestyle and can use various of organic matters as carbon, nitrogen, and energy sources. Microbe interactions may play a significant role in regulating microbe adaptation to heavy metals (Li et al., 2017). Gram-positive bacteria’s walls are effective metal chelators, and the carboxyl group of glutamic acid in peptidoglycan is the main location of metal deposition in *Bacillus subtilis*. In *Bacillus licheniformis*, teichoic and teichuronic acids are key binding sitesfor metals (Beveridge and Fyfe, 1985).Toxic chemicals, such as heavy metals, increases Extracellular Polymeric Substances (EPS) synthesis, and enzymatic processes in EPS also aid heavy metal detoxification by transforming and precipitating heavy metals in the polymeric mass (Pal and Paul, 2008).

In recent years, heavy metals have received widespread attention due to their persistence, toxicity, and accumulation in the environment (Long et al., 2021). Various metals are necessary for the growth and development of prokaryotic and eukaryotic organisms in low concentrations, however, the presence of some of these metals in higher concentrations have negative effect on these organisms. Metals have been discovered to have an adverse effect on microorganisms’ growth, morphology, and metabolic activity, resulting in lower biomass and diversity, according to several studies (Roane and Pepper, 1999). Cadmium (Cd) is highly toxic even at lower concentrations (Zhu et al., 2021). Cadmium has serious impact on the microbial growth and enzymatic activities. Cadmium can inhibit growth and respiration when present at high levels (Surowitz et al., 1984). Microbial nitrogen fixation capacity can be inhibited by about 25% by the presence of cadmium (Brookes et al., 1986).

Cadmium is abundantly present in the earth’s crust and is a non-essential and non-biodegradable heavy metal (Bruins et al., 2000; Liu et al., 2005). The increasing concentrations of cadmium in the environment are attributed to human activities, such as electroplating, mining, use of high phosphate fertilizers, and manufacturing batteries (Uqab et al., 2020). The presence of cadmium at higher concentrations also poses a threat to human health and can cause anemia, kidney damage, osteoporosis and fractures, anosmia, eosinophilia, oncogenes activation, apoptosis, Itai-Itai disease, and chronic pulmonary problems (Clemens, 2006; Suhani et al., 2021). Cadmium also exists in the blacklist agreement on Environmental protection and the World Health Organization, where it ranks among the top 10 pollutants (Bruins et al., 2000). It is pertinent to mention that Cd can easily enter the plant system and can finally find its way into the food chain (Clemens, 2006). It can also enter the transporters usually used for the uptake of essential cations, such as calcium, zinc, and iron (Shamim and Rehman, 2012). Cadmium utilization by the industries has accelerated not only biological mobilization but also the transport of this particular metal. It is one of the potent toxicants to microorganisms but at the same time, there are certain bacterial strains that are tolerant to this metal (Maldonado et al., 2010).

Cadmium is one of the most hazardous metals to living species and people, and it has biological activity in both terrestrial and aquatic animals (Haider et al., 2021; Hui et al., 2022). Cadmium enters ecosystems through a variety of human activities and emissions (Abbas et al., 2014). Because of its high mobility in contaminated soil, Cd deposition in plants in Cd-polluted soil causes substantial health risks to animals and people (Haider et al., 2021). Chlorosis and stunted development are two clearly recognizable indicators of Cd toxicity in plants, and higher toxicity restricts plant growth and leads to plant necrosis (Jali et al., 2016). Cadmium poisoning has an effect on plants by preventing carbon fixation and lowering chlorophyll concentration and photosynthetic activity (Gallego et al., 2012). Cadmium toxicity results in an excess of reactive oxygen species (ROS), which damages plant membranes and causes the death of cell macromolecules and organelles (Abbas et al., 2018).

Since heavy metals are dangerous to the environment and the living organisms (Rahman and Singh, 2018), several remediation methods have been adopted, among them remediation by using microorganisms is an effective and eco-friendly approach. Bioremediation primarily involves the use of bacteria, fungi, or yeast to clean the contaminated environment (Jiang et al., 2008). Bioremediation is a cost-effective natural process that is an alternative to incineration, catalytic destruction (Uqab et al., 2016; Irshad et al., 2021). Bioremediation is a technique for cleaning up contaminated environments by converting hazardous heavy metals into a less harmful state using bacteria or their enzymes (Ndeddy Aka and Babalola, 2016). Direct application of microorganisms with specific catabolic capabilities and/or their products, such as enzymes and biosurfactants, is a novel way to improve and augment their remediation efficacy (Le et al., 2017) So, utilizing the concept of bioremediation, we studied the role of microorganisms in sequestration of cadmium in the Saffron soils. Studies carried by Mehraj and Balkhi (2016) have suggested that Saffron soils are under the tremendous pressure of heavy metal pollution (Rafiq et al., 2008; Jan and Bhat, 2009) for which cement industries are the main source (Mehraj and Balkhi, 2016) which are adjacent to these fields and thus pose a serious threat to this economical and medicinally important crop Saffron (*Crocus sativus*). Another report from the region also suggests that pollution amounts the highest threat to saffron quality (Husaini et al., 2010). The selected site provided the best opportunity to examine and isolate the cadmium-resistant species with their respective sequestration potential.

## Materials and Methods

### Description of the Site

**Figure 1:**
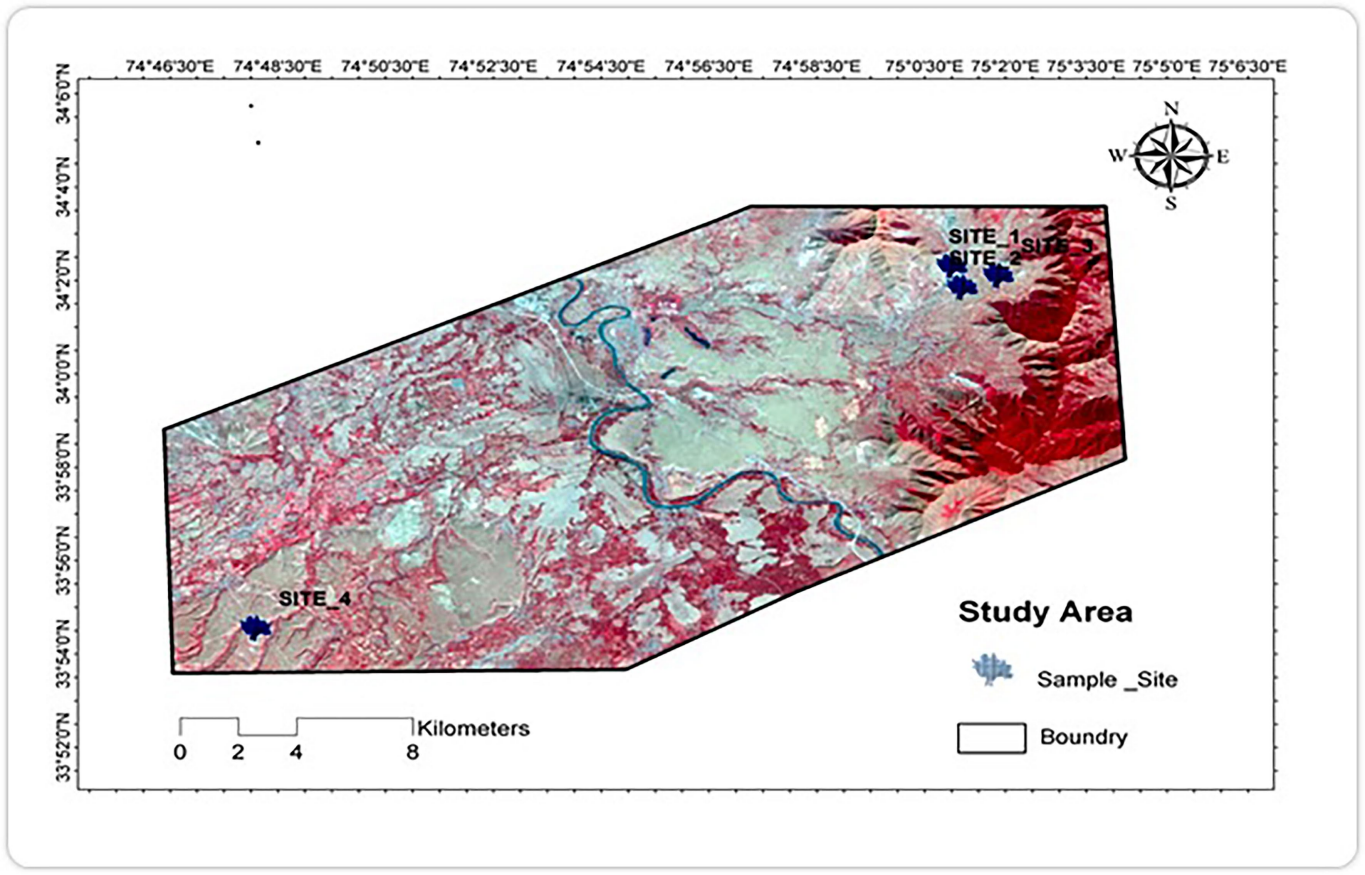
Sampling area geographical co-ordinates.

The Pampore saffron plateaus located in district Pulwama of Jammu & Kashmir were selected for the collection of soil samples, as the proximity of the town is dominated by a large number of cement factories that discharge heavy metal-containing effluents resulting in high accumulation levels of heavy metals in the surrounding soil. Composite soil samples were collected from a depth of 15 cm for the current analysis. Three separate locations (Figure 1) identified in this area according to the degree of influence were chosen to investigate cadmium resistant bacteria with the control site from the non-industrial zone.

### Soil Sample Processing

The collection of soil samples was done seasonally from the selected locations for two and a half years (10 seasons). From the various sites, the replicates of the soil samples were collected in equal proportions (approx. 10 g). The sample was mixed systematically and subjected to segregation of cadmium-resistant bacteria by using the standard protocol (Jett et al., 1997; APHA, 2005).

### Isolation and Enumeration of Bacteria

Serial dilution of soil samples with normal saline was performed for bacterial confinement accompanied by immediate direct plate (Jett et al., 1997; APHA, 2005; Chen et al., 2005) and pure culture technique (Olofsson et al., 2007). About 0.1 ml of inoculum was subsequently dispersed from the tubes containing serial dilution (Ben-David and Davidson, 2014) on petri-plates containing the nutrient agar and Luria-Bertani (LB) agar. The plates were incubated for about 24 h at a temperature of 37°C (APHA, 2005). The number of colony-forming units (CFU/g) of soil was then used to get the viable count of bacteria.

### Identification

The bacterial isolates were identified using morphological characteristics, sequencing of the 16S rRNA gene, and Matrix-assisted laser desorption ionization time-of-flight mass spectrometry (MALDI-TOF MS) identification.

### Morphological Approach

To analyze, macro- as well as micro-morphological features of bacteria, incubation of media plates, were done for 1–3 days at 37°C. Following which the bacteria were characterized on the basis of colony characteristics *viz*. colony size, shape, elevation, color, opacity, and margins. The morphological description of the bacteria was carried out using Gram’s staining method followed by direct microscopy (100x Olympus IX 71 fluorescence microscope).

### Identification Based on MALDI-TOF MS

The Matrix-assisted laser desorption ionization time-of-flight mass spectrometry (MALDI-TOF MS) technique was used to identify bacteria using the Ethanol-formic acid extraction process (Alatoom et al., 2011; Rahi et al., 2016).

### 16S rRNA Gene Sequencing

GenElute bacterial Genomic DNA kit (Sigma) was used to extract and purify DNA according to manufacturing protocol followed by amplification of the 16S rRNA gene using universal bacterial primers forward primers [(27F) 5′AGAGTTTGCCTGCTCAG-3′] and reverse primers [(1429R) 5′-GGGTTACCTGACTGACTTT-3′; Chatterjee et al., 2012]. For amplification of the 16S rRNA gene, Ependroff thermal cycler with correct conditions was used. The PCR products obtained were purified and sequenced at AgriGenome Kerala and the National Center for Microbial Resource (NCMR) Pune using Sanger’s dideoxy sequencing system. The sequences obtained were further evaluated for similarity searches against the sequences available in NCBI. The Phylogenetic tree was also constructed using Mega X software.

### Cadmium Tolerance and MIC

Cadmium tolerance was evaluated by the preparation of a stock solution (1,000 ppm) of cadmium chloride (Himedia) screened *via* a 0.45 μm pore size syringe filter to eliminate any bacterial contamination. To determine the MIC, various concentrations were prepared from the stock solution using the Muller Hinton Broth as per the existing protocol (Yilmaz, 2003; Wiegand et al., 2008). Before dilution of broth, all the isolates were re-cultured and put into 1X PBS solution, maintaining the OD of 1, which equals to the McFarland standard and comprises of 3.0 × 10^8^ colonies (McFarland, 1907). Hence, considered the lowest possible inhibiting concentration as MIC.

### Sequestration Potential of Cadmium Tolerant Isolates

The sequestration potential of isolates tolerant to cadmium was carried out using standard methods (Shin et al., 2012; Alboghobeish et al., 2014), and analysis of cadmium was performed according to methods given by APHA (2005). Muller Hinton broth (with 100 ppm cadmium) was placed in the shaking incubator (180 rpm) at 37°C for about 48 h for metal sequestration and after every 6-h, 5 ml of the sample was extracted from the flask and centrifuged at 10,000 rpm for 5 min. For the acid digestion of supernatant two acids *viz*., sulphuric acid and nitric acid were used followed by the determination of metal concentration in percentage by Atomic Absorption Spectrometer (AAS Perkin Elmer Germany).

## Results and Discussion

### Characterization of Bacteria

Enumeration of the bacterial colonies obtained on the petri-plates was carried using a digital colony counter, and the colony forming units (CFUs/g) were used to assess the bacterial load. In summer seasons, bacterial load was found to be in elevated concentration as compared to winter seasons. In the successive years of study, we observed an overall increase in bacterial load during all four seasons of the year. In the three sampling sites, the CFU/g was found to vary between 8 × 10^3^ and 9.5 × 10^4^ CFU/g. Out of the 72 bacterial isolates, the percentage of Gram-negative was found to be 35.5%, whereas the percentage of Gram-positive was 64.5%. However, it was revealed by the taxonomical classification that the most dominant family among the isolated bacteria was *Bacillaceace* (43.24%) followed by other families like *Enterobacteriaceae* (21.62%) *Planococcaceae*(8.10%), *Microbacteriaceae* (8.10%), *Pseudomonadaceae* (5.40%), Staphylococceaceae (5.40%), and *Aeromonadaceae* (2.70%).

### Molecular and MALDI-TOF MS Characterization

**Figure 2:**
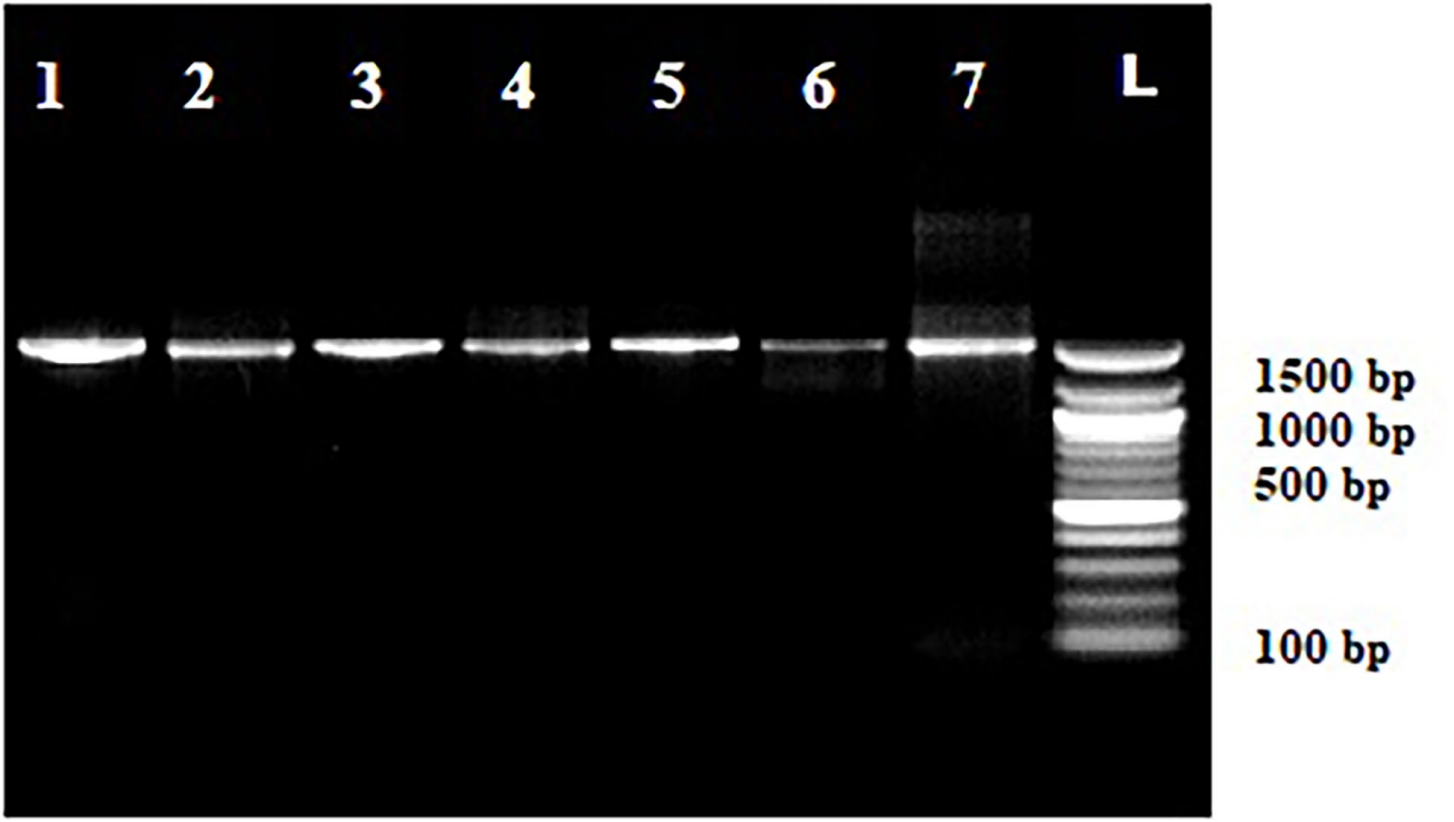
Amplification of 16S-rRNA in cadmium tolerant species. Lanes, 1 *Pseudomonas putida*, Lanes, 2 *Bacillus pumilus*, Lanes, 3 *Klebsiella pneumoniae*, Lanes, 4 *Pseudomonas mandelii*, Lanes, 5 *Pseudomonas avellanae*, Lanes, 6 *Enterobacter kobei*, and Lanes, 7 *Staphylococcus equorum*. Lanes, 8 Ladder (100 bp Fermentas).

**Figure 3:**
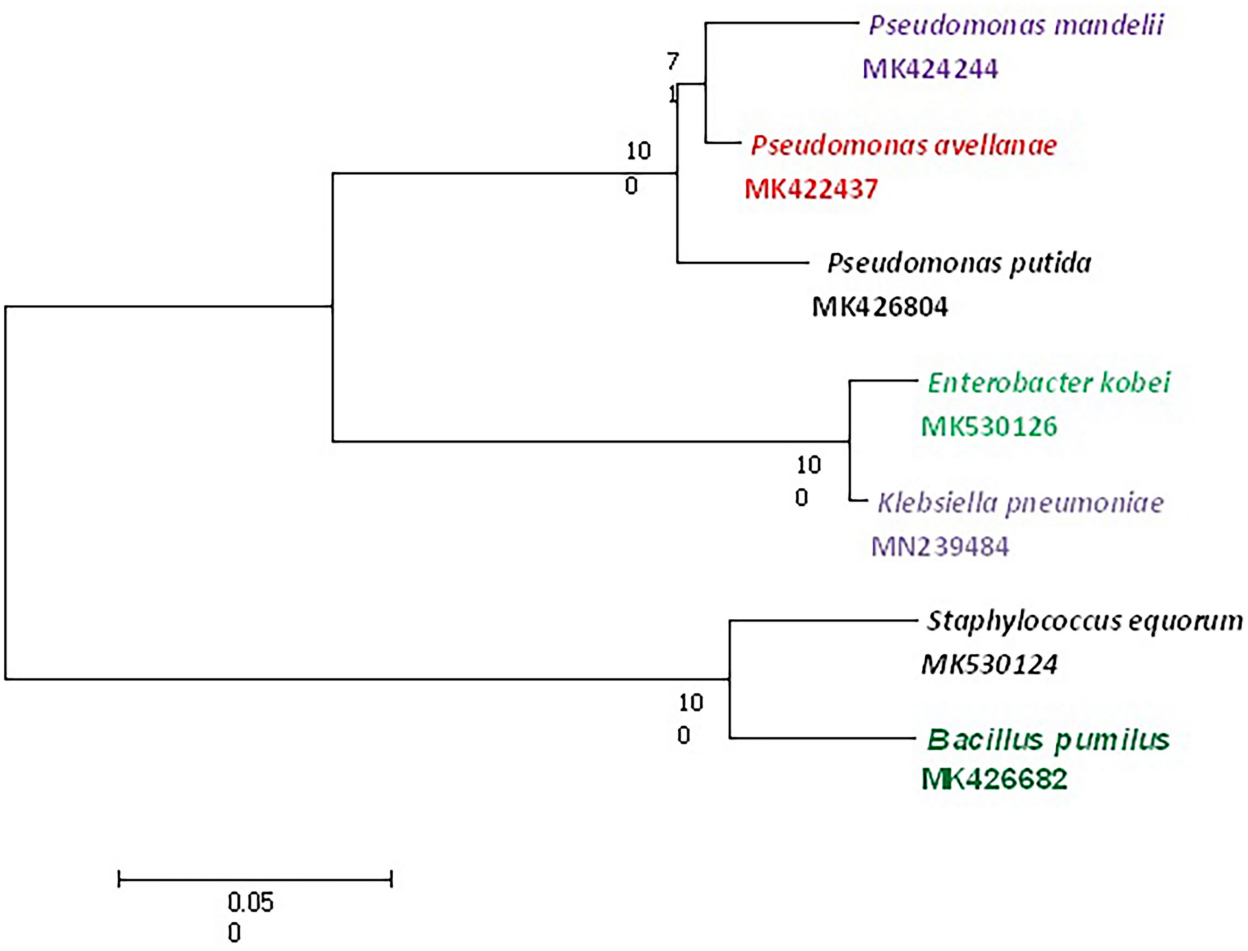
Phylogenetic analysis of the cadmium tolerant isolates obtained in this study.

Out of 72 total isolates only seven bacterial species identified by MALDI-TOF MS and 16S rRNA gene sequencing were found to be tolerant to cadmium (Figures 2, 3) *viz*., *P. putida, B. pumilus, Klebsiella pneumoniae, P. mandelii, P. avellanae, E. kobei* and *S. equorum*.

Based on the Tamura-Nei model (Tamura and Nei, 1993) evolutionary history showed the greatest log probability of −2713.79 with the proportion of trees in which the related taxa clustered together next to the branches. Analysis involving seven nucleotide sequences was applied to scale, measuring branch lengths in the amount of replacements per site, and eliminating all positions containing gaps and missing information. In addition, evolutionary analysis in MEGA X disclosed a total of 800 locations in the final dataset. In addition, phylogenetic analysis was performed for the metal tolerant species using the maximum likelihood method. The evolutionary history was inferred by using ML and Kimura two-parameter to look for clustering of clads in different tress. The bootstrap consensus tree inferred from 1,000 replicates is taken to correspond to the evolutionary history of the taxa analyzed (Felsenstein, 1985). The percentage of replicate trees in which the associated taxa clustered together in the bootstrap test is shown next to the branches. Evolutionary analysis was conducted in MEGA X and involved seven nucleotide sequences having a total of 800 positions in the final data set (Kumar et al., 2018).

### Matrix-Assisted Laser Desorption Ionization Time-of-Flight Mass Spectrometry

**Figure 4:**
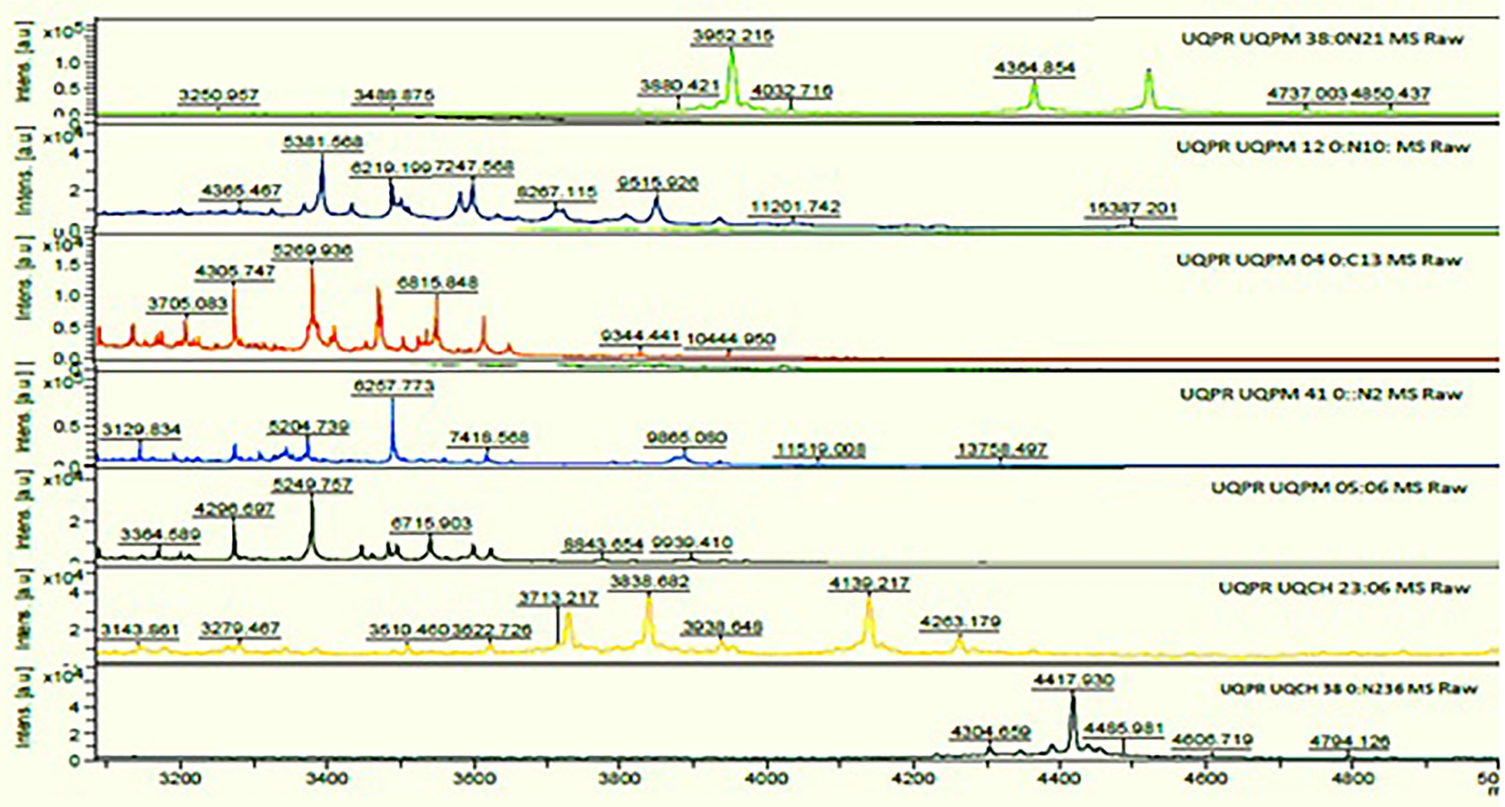
MS spectra of cadmium tolerant isolates on matrix-assisted laser desorption ionization time-of-flight mass spectrometry (MALDI-TOF MS).

All the cadmium-resistant bacteria isolates were evaluated and thus analyzed using a linear MALDI-TOF mass spectrometer analysis (Bruker Daltonics, Germany with Bruker Biotype 2.0 software and library). The criterion score that was used for identification was as per the recommendations of the manufacturer. The isolates presenting a score of ≥2.000 reveal identification up to species-level, a score between 1.700 and 1.999 illustrate identification up to genus level, and a score of <1.700 revealed no identification. The MS spectra thus produced (Figure 4) and the extracted protein identified the isolates as *P. putida, B. pumilus, Klebsiella pneumonia, P. mandelii, P. avellanae, E. kobei*, and *S. equorum*.

Based on Figure 4 the standard mass spectra obtained from the MALDI-TOF-MS analysis for cadmium metal tolerant species are: *K. pneumoniae* showing nine spectra in the ion range at m/z 3250.957–4850.437; *P. putida*, six spectra in the ion range at m/z 3705.083–10444.950; *B. pumilus*, eight spectra in the ion range at m/z 4365.567–15387.201; *P. avellanae*, nine spectra in the ion range at m/z 3143.861–4263.179; *P. mandelii* showed seven spectra in the ion range at m/z 3129.834–13758.497; *E. kobei*, eight spectra in the ion range at m/z 4365.467–15397.201 and *S. equorum* showed five spectra in the ion range at m/z 4304.659–4794.126. MALDI-TOF-MS profile analysis revealed the presence of species-specific ion signals.

Both the sequencing of the 16Sr RNA gene and the MALDI-TOF MS analysis gave identical identification results of isolates hence, it can be inferred that both the techniques are reliable and can be used for the identification of bacteria.

### Metal Tolerance

**Figure 5:**
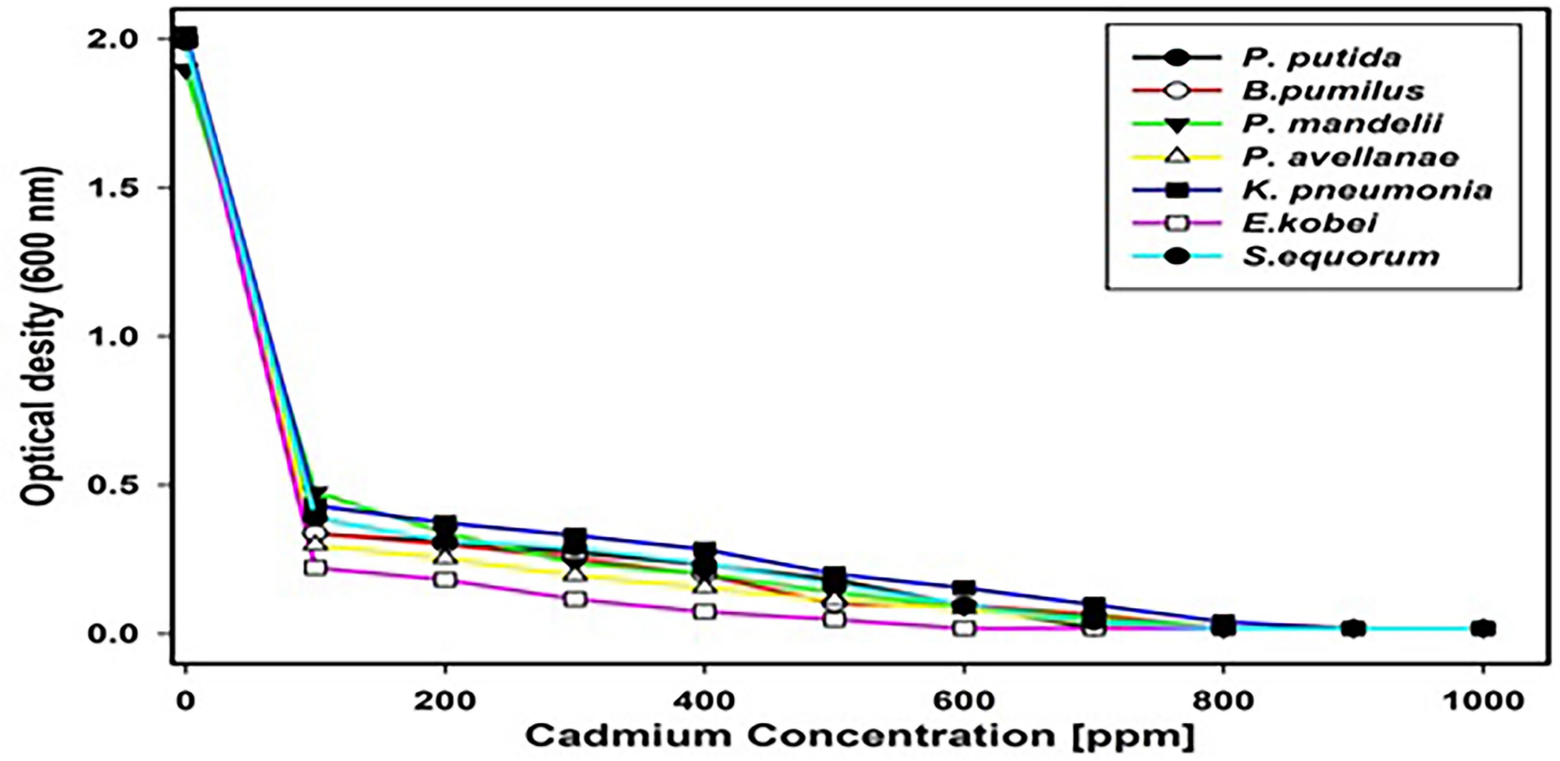
Optical Density (OD) values indicating minimum inhibitory concentrations (MICs) of isolates resistant to cadmium.

The tolerance of all isolates against cadmium and the subjective MIC (Figure 5.) determined for the tolerant isolates showed that *K. pneumoniae* has the highest (900 ppm) and *E. kobei* the lowest (600 ppm) tolerance against cadmium.

### Sequestration Potential

**Figure 6:**
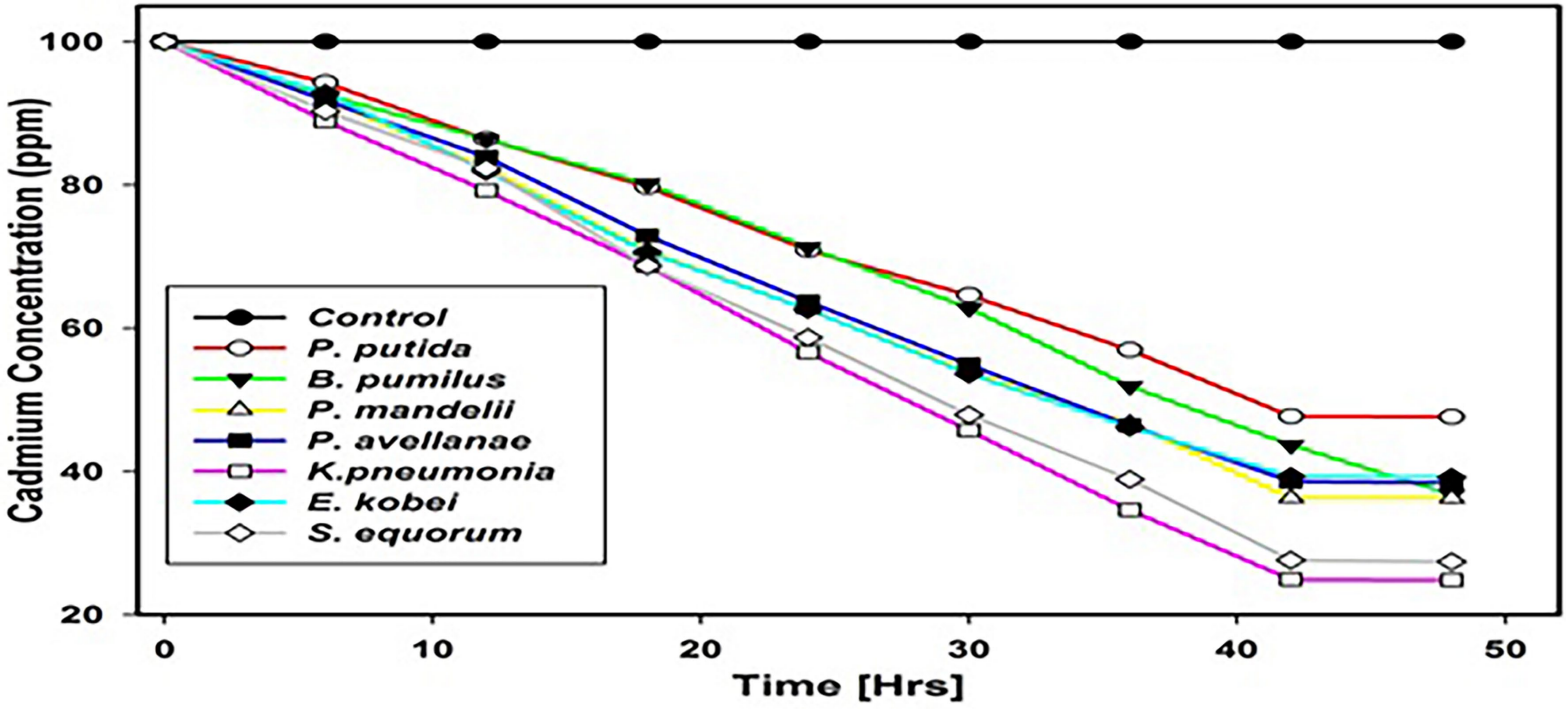
Sequestration potential of cadmium-tolerant metal isolates in ppm.

For all cadmium tolerant isolates, the sequestration potential was found to vary significantly after 48 h for each isolate and *K. pneumoniae* showed the highest sequestration potential of 75.2% among the cadmium tolerant species, whereas *P. putida* showed the lowest sequestration potential of 52.4% (Figure 6).

Being an industrial area and encircled by about 18 cement factories, Pampore is facing the serious problem of cement pollution. The calcination of raw materials in a kiln produces clinker, which is used to make cement. Thermal activities, such as iron manufacturing, fossil fuel burning, and cement production, all emit cadmium into the atmosphere (Işıklı et al., 2006). The geographic location of this town adds more problems as the mountains surrounding this area confine the cement dust within the area. Heavy metals discharged through these factories pose a serious threat to saffron, which is an economically important cash crop of the Kashmir valley (Rafiq et al., 2008; Uqab et al., 2020). Due to the non-biodegradable nature and bioaccumulation in the system, heavy metal pollution has become a major concern worldwide in recent years. Such knowledge may be used to assess the effects of environmental stress on the ecosystem and other variables like deforestation, agriculture, and climate change (McGuire and Treseder, 2010; Vos et al., 2013; Macdonald and Singh, 2014). The increasing population surely has contaminated the essential elements of life which include air water and land (Gadd, 2010). The major warning to human life is the bioaccumulation of heavy metals in the environment. Cadmium being the major contaminant, if found in the environment, can be extremely poisonous to humans, animals, plants as well as to microbes as it has the potential to damage the cell membranes, alter the DNA structure and affect the enzyme specificity (Torsvik and Øvreås, 2002; Dynarski and Houlton, 2018). A total of 72 bacterial species have been isolated in the current study from the saffron fields of Kashmir valley. However, the presence of these diverse species present in the soil can be due to the availability of a large number of nutrients in these soils, which fosters the growth and developmental conditions of such microbes (Pommier et al., 2018). The heavy metal resistant species of microbes occur naturally in environments that are contaminated with metals and such environments create a selection pressure to facilitate their propagation and existence (Rahman and Singh, 2018). Metal resistance is a general phenomenon in several microorganisms that own various mechanisms like a comprehensive delay for adapting to the heavy metal ions (Gadd, 2010). The soil harbors a huge number of microbial communities and their structures fluctuate greatly among distinct settings (Macdonald and Singh, 2014). Themicrobial communities play a remarkable role in determination of various functions of soil including carbon turnover, nitrogen mineralization, and pest population (Torsvik and Øvreås, 2002; Macdonald and Singh, 2014).

Owing to a large number of cement industries and rigorous use of fertilizers (Husaini et al., 2010), the saffron soils are currently facing a serious threat mostly because of the presence of heavy metals (Mehraj and Balkhi, 2016). In the current study, we isolated seven cadmium resistant isolates which were identified as *P. putida, B. pumilus, K. pneumoniae, P. mandelii, P. avellanae, E. kobei*, and *S. equorum*. These bacteria are considered as “metallophiles” ascribed to their production of Metallothioneins (MTs), which are involved in protecting these cells from toxic metal effects (Blindauer, 2011; Jarosławiecka and Piotrowska-Seget, 2014). The MIC results revealed that Cd tolerant bacteria, *K. pneumonia* exhibited the highest range of tolerance (900 ppm) while *E. kobei* showed the lowest range (600 ppm). Several studies had shown that bacteria have the effectiveness to remove Cd from contaminated environments. Similar to our findings bacterial species like *Pseudomonas aeruginosa* and *Rhodobacter sphaeroides* have shown promising results in Cd removal in fixed temperatures as reported by Bai et al. (2008), these results support our findings for isolation of cadmium tolerant bacteria (Bai et al. 2008). Zhou et al. have also isolated *Pseudoalteromonas* sp., from the deep-sea that have shown efficacy for Cd removal *via* biosorption and intracellular uptake from the liquid media and the same mechanism can be attributed to the bacterial species like *P. putida* and *P. mandelii* of the current report (Zhou et al., 2013). Cd tolerant bacterial species isolated from the contaminated environments possess the intrinsic mechanism to combat the toxicity of the metal and have significant potential for removal of the metal from the environment (Zhou et al., 2013). Ahmad et al. (2014) reported that *Klebseilla* sp. tends to decrease the Cd concentration which shows a complete corroboration with our results. In another study, *Bacillus* sp., from mining soils with cadmium tolerance have shown Cd removal property by converting soluble Cd into insoluble carbonate crystal (Zhao et al., 2017). In the same way, Shamim and Rehman (2012) have isolated *Klebseilla pneumoniae* with a tolerance limit of up to 1,500 ppm which even though is higher than the MIC values of our results but follows a similar trend. Bacterial species like *Burkholderia cenocepaci* and *Thalassiosira wessflogii* can tolerate high concentrations of Cd and can also use cadmium as a nutrient (Shamim and Rehman, 2012). Chovanová et al. (2004) have revealed that bacterial isolates like *Klebsiella planticola, Pseudomonas fluorescens, P. putida*, and *Serriatia liquefaciens* that have been isolated from the sewage sludge possess significant potential for Cd removal (Chovanová et al., 2004). A study carried out by Xu et al. (2015) also revealed that *Penicillium chrysogenum* XJ-1 has a high tolerance of Cd and can decrease the concentration *via* the same processes like biosorption, intracellular uptake, or metal sequestration. Further our results of bacteria for cadmium tolerance are also supported by the main mechanism present in the microorganisms to detoxify cadmium through the binding of polythiols. This mechanism is not restricted to cadmium only but also associated with other heavy metals. Furthermore, our data can also be extensively explained based on the study reported by Abbas et al. (2014) according to which resistance in the microorganism for the cadmium is divided into two mechanisms; active and passive. The active mechanism includes efflux pump, volatilization, precipitation, oxidation, intracellular accumulation, metallothioneins, and rhamnolipid while as passive mechanism includes binding to cell wall, extracellular complexation, EPS, and siderophores (Abbas et al., 2014).

Sequestration potential of the isolates indicated *E. kobei* showed the highest potential of 75.3% after 48 h, while *P. putida* showed the least sequestration potential of 52.4%. Uptake of the metals by the microorganism is based upon the physiology involving two mechanisms; passive mechanism and active mechanism. The passive mechanism does not need consumption of energy and is also known as the metabolic-independent mechanism (Haq et al., 1999; Ahluwalia and Goyal, 2007). This mechanism removes metals from the solution *via* complexation with the help of extracellular biological chelates or by binding metals to the cell surfaces (Ahalya et al., 2003). Microorganisms secrete EPS that are responsible for the complexation of the metals. Adsorption processes are involved in the binding of metals with the cell wall and these processes comprise ionic, physical, and chemical adsorption. In case of the ionic processes, peptidoglycan, techoic acid, alginates, or polysaccharides at the surface of microorganisms exchange their counter ions against divalent metal ions in the solution. While in case of the physical adsorption, weak forces like electrostatic force and Van-der-Waals force helps in binding metal ions to the surface of microorganisms (Gavrilescu, 2004). However, the intracellular cadmium mechanisms have been categorized into three different phases *viz*. binding, metabolism, and precipitation of cadmium phosphates (Naik et al., 2018).The active mechanism that need consumption of energy in the form of ATP is also known as metabolic dependent mechanism and is exclusively found in micro-organisms. Metal sequestration processes by extra cellular sequestration and intracellular sequestration play important role in the removal of metal from the contaminated environs (Mardanov et al., 2016). In case of extra cellular sequestration, microbes produce metabolites like oxalate, sulfur, glutathione, and phosphate. These metabolites have property to bind metals (Lima e Silva et al., 2012).

Thus, to detoxify and eradicate the cadmium from the affected areas, the cadmium resistant property of the isolated bacterial species could prove to be advantageous.

## Conclusion

Matrix-assisted laser desorption ionization time-of-flight mass spectrometry and 16S rRNA sequencing documented seven cadmium tolerant species of bacteria (*P. putida, B. pumilus, Klebsiella pneumonia, P. mandelii, P. avellanae, Enterobacter Kobe*, and *S. equorum*). In the present investigation, *K. pneumoniae* showed the excellent ability for cadmium sequestration, i.e., 75.2%, and showed a tolerance level of 900 ppm. The other bacterial species tolerant to cadmium have shown promising results in tolerance and sequestration as well. The primary conclusion depicts the cadmium tolerance among different isolated bacterial species collected from the saffron soils of Kashmir valley and further highlighting their role in Cd sequestration. Besides, detoxification and elimination of the heavy metals from soils contaminated with cadmium, the cadmium-resistant property might prove to be beneficial, thereby offering possible alternatives for the process of bioremediation.

## Data Availability Statement

The data presented in the study are deposited in GenBank repository and accession numbers are MK424244, MK422437, MK530126, MN239484, MK530124, MK426682, and MK426804. All the data has been released by the GenBank.

## Author Contributions

BU: writing—original draft, writing—review and editing, data curation, and formal analysis. RN: concept design, data curation, formal analysis, writing, review, and editing. BG: writing—review and editing. PR: review and editing. All authors contributed to the article and approved the submitted version.

## Conflict of Interest

The authors declare that the research was conducted in the absence of any commercial or financial relationships that could be construed as a potential conflict of interest.

## Publisher’s Note

All claims expressed in this article are solely those of the authors and do not necessarily represent those of their affiliated organizations, or those of the publisher, the editors and the reviewers. Any product that may be evaluated in this article, or claim that may be made by its manufacturer, is not guaranteed or endorsed by the publisher.
